# Genotypes and Pathogenicity of Cellulitis Isolates Reveal Traits That Modulate APEC Virulence

**DOI:** 10.1371/journal.pone.0072322

**Published:** 2013-08-19

**Authors:** Nicolle Lima Barbieri, Aline Luísa de Oliveira, Thiago Moreira Tejkowski, Daniel Brisotto Pavanelo, Débora Assumpção Rocha, Letícia Beatriz Matter, Sidia Maria Callegari-Jacques, Benito Guimarães de Brito, Fabiana Horn

**Affiliations:** 1 Departamento de Biofísica, Universidade Federal do Rio Grande do Sul, Porto Alegre, Rio Grande do Sul, Brazil; 2 Instituto de Pesquisas Veterinárias Desidério Finamor, Eldorado do Sul, Rio Grande do Sul, Brazil; 3 Departamento de Ciências da Saúde, Universidade Regional Integrada do Alto Uruguai e das Missões, Santo Ângelo, Rio Grande do Sul, Brazil; 4 Departamento de Estatística, Universidade Federal do Rio Grande do Sul, Porto Alegre, Rio Grande do Sul, Brazil; Centre National de la Recherche Scientifique, Aix-Marseille Université, France

## Abstract

We characterized 144 *Escherichia coli* isolates from severe cellulitis lesions in broiler chickens from South Brazil. Analysis of susceptibility to 15 antimicrobials revealed frequencies of resistance of less than 30% for most antimicrobials except tetracycline (70%) and sulphonamides (60%). The genotyping of 34 virulence-associated genes revealed that all the isolates harbored virulence factors related to adhesion, iron acquisition and serum resistance, which are characteristic of the avian pathogenic *E. coli* (APEC) pathotype. ColV plasmid-associated genes (*cvi/cva, iroN, iss, iucD, sitD, traT, tsh*) were especially frequent among the isolates (from 66.6% to 89.6%). According to the Clermont method of ECOR phylogenetic typing, isolates belonged to group D (47.2%), to group A (27.8%), to group B2 (17.4%) and to group B1 (7.6%); the group B2 isolates contained the highest number of virulence-associated genes. Clonal relationship analysis using the ARDRA method revealed a similarity level of 57% or higher among isolates, but no endemic clone. The virulence of the isolates was confirmed *in vivo* in one-day-old chicks. Most isolates (72.9%) killed all infected chicks within 7 days, and 65 isolates (38.1%) killed most of them within 24 hours. In order to analyze differences in virulence among the APEC isolates, we created a pathogenicity score by combining the times of death with the clinical symptoms noted. By looking for significant associations between the presence of virulence-associated genes and the pathogenicity score, we found that the presence of genes for invasins *ibeA* and *gimB* and for group II capsule *KpsMT*II increased virulence, while the presence of *pic* decreased virulence. The fact that *ibeA*, *gimB* and *KpsMT*II are characteristic of neonatal meningitis *E. coli* (NMEC) suggests that genes of NMEC in APEC increase virulence of strains.

## Introduction

Extraintestinal infections caused by avian pathogenic *Escherichia coli* (APEC) include omphalitis in embryos, salpingitis in laying hens, respiratory tract infections, and cellulitis [1]. Cellulitis is one of the most prevalent extraintestinal infections caused by APEC in broiler chickens, and is characterized by the presence of subcutaneous fibrinonecrotic plaques and inflammation of the overlying chicken skin, resulting in rejection of part or all of the carcasses at processing [2], [3], [4], [5]. In Brazil, cellulitis lesions are estimated to cause the loss of 0.14 to 1.4% of poultry meat production [6], leading to losses of at least 18 thousand tons of meat in 2011 [7]. Depending on the virulence of the strain, the localized infections may become systemic [8].

The virulence genes that permit certain intestinal commensal *E. coli* to become APEC and infect extraintestinal sites include those encoding for the adhesins type 1 fimbriae and temperature-sensitive haemagglutinin (Tsh), iron-scavenging systems and the protectin Iss [9]. Most of these genes are often carried on Colicin V (ColV) or other large plasmids, and are thought to enable APEC strains to adhere to host tissues, survive within host fluids and resist host immune defenses [10], [11], [12], [13]. Different APEC strains may have unique combinations of different virulence factors that have similar functions with regards to disease establishment. Despite our knowledge about the APEC pathotype, we still depend on *in vivo* assays to make sure that an *E. coli* isolate is able to cause an extraintestinal infection [14] and to determine the degree of virulence of the strain [8], [15].

By genotyping a North American collection of APEC strains of known virulence in one-day-old chicks [16], Johnson et al. [17] identified five ColV-associated genes that distinguish an APEC from a non-pathogenic strain. Schouler et al. [14] combined the virulence genotyping of a large European collection with *in vivo* virulence tests in one-day-old chicks to identify four groups of virulence genes associated with APEC. The virulence traits proposed by Johnson et al. [17] and Schouler et al. [14] represent potentially efficient ways for screening APEC strains occurring during poultry production. Neither work, however, allows the prediction of the degree of virulence of an APEC isolate.

In the present work we have genotyped 144 cellulitis isolates from broiler chickens in Southern Brazil and tested them for virulence in one-day-old chicks. We attributed a pathogenicity score to each isolate, which takes into account not only the number of deaths within 7 days, but also the clinical symptoms manifested before death and how quickly the infection kills birds. The pathogenicity score represents an improvement on the lethality test using the same number of animals, and may help to discriminate between different degrees of APEC virulence. We also characterized the isolates in terms of their resistance to 15 antimicrobial agents, their *E. coli* reference collection (ECOR) phylogenetic typing, and their clonal relationships.

## Materials and Methods

### Ethics statement

All animal experiments were approved by the Biosafety Committee of the Instituto de Pesquisas Veterinárias Desidério Finamor (CIB 004/08), and chickens were euthanized according to animal welfare norms.

### Bacterial strains

One hundred and forty-four *E. coli* isolates were obtained between October 2006 and March 2007 from severe cellulitis lesions in 7-week-old broiler chickens at the time of their slaughter. The isolates were collected from different poultry flocks in 65 distinct farms in various locations within the southern Brazilian state of Paraná (PR). Farms could have more than one flock of broiler chickens: in order to ensure diversity of the strains, we collected only one isolate per flock. Biochemical tests (triple sugar iron, urease and MacConkey) were performed to confirm that all isolates were *E. coli* [18]. All strains were stored at –80°C in Luria-Bertani (LB) broth with 20% glycerol until they were needed.

### Antibiotic resistance in APEC

The antimicrobial susceptibility of all APEC isolates was examined using the disc diffusion test according to the Clinical and Laboratory Standards Institute guidelines [18], using *Escherichia coli* strain ATCC 25922 as a control. The 15 antimicrobial agents tested were: ampicillin (10 U), bacitracin (10 U), cephalothin (30 µg), ceftiofur (30 µg), ciprofloxacin (5 µg), chloramphenicol (30 µg), enrofloxacin (5 µg), gentamicin (10 µg), neomycin (30 µg), nitrofurantoin (300 µg), norfloxacin (10 µg), tetracycline (30 µg), sulphonamides (300 µg), trimethoprim (5 µg) and a combination of sulphonamides and trimethoprim (23.7 µg plus 1.3 µg). All antimicrobial discs were from CEFAR (São Paulo, Brazil). These antimicrobials were selected because they are, or were previously, employed in the poultry industry as growth promoters, for disease prevention and/or for treatment. The breakpoints were obtained from CLSI 2009 [18] for all antimicrobials, except for ampicillin, cephalothin, chloramphenicol and enrofloxacin [19], ceftiofur [20] and neomycin [21].

### DNA extraction

Bacterial DNA was obtained from whole organisms by boiling [22]. The extracts were stored at 4°C, and the supernatants were used as templates for gene amplification.

### Multiplex polymerase chain reactions

The presence of 33 virulence-associated genes in the isolates (Table 1) was investigated using multiplex polymerase chain reactions as described [23] with a few modifications, as outlined in [22].

**Table 1.** Prevalence of VAGs in cellulitis isolates as detected by PCR.

**Table:** Table 1. Prevalence of VAGs in cellulitis isolates as detected by PCR.

Gene(s) or operon	Description	Size	n	% (*n* = 144)
**Adhesins**				
afa/draB	Afimbrial/Dr antigen-specific adhesin	809 pb	0	0
csgA	Cryptic curlin subunit	200 pb	144	(100.0)
crl	Curli fiber gene	249 pb	127	(88.2)
fimC	Type 1 fimbriae (D-mannose specific adhesin)	496 pb	132	(91.7)
hra	Heat-resistant agglutinin	540 pb	66	(45.8)
iha	Iron-regulated-gene-homologue adhesin	608 pb	18	(12.5)
papC	Pilus associated with pyelonephritis	500 pb	44	(30.5)
sfa/focCD	S fimbriae (sialic acid-specific) and F1C fimbriae	1222 pb	6	(4.2)
tsh^1^	Temperature sensitive hemagglutinin	823 pb	96	(66.6)
mat	Meningitis associated and temperature regulated fimbriae	898 pb	101	(70.1)
**Iron acquisition**				
chuA	Heme receptor gene (E. coli haem utilization)	278 pb	83	(57.6)
fyuA	Ferric yersinia uptake (yersiniabactin receptor)	773 pb	67	(46.5)
ireA	Iron-responsive element	384 pb	100	(69.4)
iroN^1^	Catecholate siderophore (salmochelin) receptor	846 pb	110	(76.4)
irp2	Iron repressible protein (yersiniabactin synthesis)	286 pb	96	(66.6)
iucD^1^	Aerobactin synthesis	710 pb	117	(81.2)
sitD chr.	Salmonella iron transport system gene	553 pb	21	(14.6)
sitD ep.^1^	Salmonella iron transport system gene	1032 pb	100	(69.4)
**Protectins/Serum resistance**				
cvi/cva^1^	Structural genes of colicin V operon (Microcin ColV)	597 pb	83	(57.6)
iss^1^	Increased serum survival	309 pb	114	(79.2)
neuC	K1 capsular polysaccharide	675 pb	31	(21.5)
kpsMT II	Group II capsule antigens	269 pb	53	(36.8)
ompA	Outer membrane protein	918 pb	137	(95.1)
traT^1^	Transfer Protein	429 pb	129	(89.6)
**Toxins**				
astA	EAST1 (heat stable cytotoxin associated with enteroaggregative E. coli)	110 pb	48	(33.3)
cnf1/2	Cytotoxic necrotizing factor	445 pb	0	0
sat	Secreted autotransporter toxin	666 pb	2	(1.4)
vat	Vacuolating autotransporter toxin	980 pb	51	(35.4)
hlyA	Hemolysin A	350 pb	1	(0.7)
**Invasins**				
gimB	Genetic island associated with newborn meningitis	736 pb	14	(9.7)
ibeA	Invasion of brain endothelium	341 pb	30	(20.8)
tia	Toxigenic invasion locus in ETEC strains	511 pb	26	(18.0)
Miscellaneous				
pic	Serin protease autotransporter	410 pb	38	(26.4)
malX	Pathogenicity-associated island marker	921 pb	11	(7.6)

1 Genes associated with large virulence plasmids in APEC, such as pAPEC-O2-ColV [NC_007675], pTJ100 [AY553855], pAPEC-O1-ColBM [NC_009837], pAPEC-O1-R ( NC_009838), pAPEC-O2-R NC_006671, pAPEC-O103-ColBM NC_011964, pAPEC-1 NC_011980.1.

### PCR-based classification into "ECOR" phylogenetic groups

All 144 isolates were classified using the multiplex PCR-based phylogenetic typing method of Clermont et al. [24], which groups strains into the four main phylogenetic groups shown in the reference strains in the ECOR collection [25]. Reactions were performed in a GenePro Thermal Cycler (Bioer Technology, China) as follows: denaturation for 4 min at 94°C, 30 cycles of 5 s at 94°C and 10 s at 59 °C, and a final extension step of 5 min at 72°C.

### Phylogenetic analysis

Genetic data was obtained using the Amplified Ribosomal DNA Restriction Analysis (ARDRA) method [26]. This method is based on the variability of the ribosomal 16S-23S intergenic spacer region (ISR), which is well-distributed among isolates and has slow rates of mutation, and hence is considered useful for measuring intra-species diversity [26], [27]. The ISR region was amplified and digested with restriction enzymes (*RsaI*, *HinfI* or *TaqI*) as previously described [22]. ARDRA restriction fragment length polymorphism profiles were analyzed by eye, and were converted into two-dimensional binary matrices according to the following criteria: 1 if a band was present, and 0 if it was absent. A matrix of distances was calculated and a dendrogram was produced using the NTSYS-pc program (version 2.0, Exeter Software, Setauket, NY). The Unweighted Pair Group Method with the Arithmetic Mean (UPGMA) was used.

### Lethality and pathogenicity tests

Groups of 10 one-day-old Cobb female chicks were inoculated subcutaneously with 100 µL (10^8^ CFU) of an overnight culture containing ∼10^9^ CFU/mL of each APEC isolate. A control group was inoculated with BHI broth. The animals were observed at 12 h intervals over 7 days, with all deaths being recorded. The lethality score (LS) was calculated according to the number of animals that died within this period with a range from 0 (no animal died) to 10 (all animals died) [2], [28]. At 7 days post-infection, surviving chicks were killed by cervical dislocation, and clinical scores were recorded. Times of death and clinical scores were combined to give pathogenicity scores (PS), as described by Barbieri et al. [22]. Briefly, we performed postmortem examinations after chick deaths, looking for evidence of airsacculitis (A), pericarditis (P), perihepatitis (Ph), peritonitis (Pe) and cellulitis (C). The presence of a lesion was given the value 1, and its absence, the value 0. Pathogenicity scores (PS) were calculated from the equation PS  =  (TD x 5) + P + Pe + Ph + A + C, in which TD corresponds to the day of chick death, which has a value of 1 if the animal dies on the first day, and is reduced by 0.14 for each day that the animal survives up to day 7, which has the value 0. According to this equation, the PS can vary from 0 to 10.

Animals that died on the first day after inoculation had their livers dissected, homogenized and plated on lactose-containing MacConkey agar to identify *E. coli*; a PS  =  10 score was attributed to these animals. The PS for each strain was calculated as the median PS for the 10 chicks infected with that particular strain.

### RNA purification and quantitative real-time RT-PCR

*E. coli* strains PR001, PR013, PR017 and PR034 were grown overnight in BHI media. RNA from these strains was stabilized by RNAprotect Bacterial Reagent (QIAGEN) and extracted using an RNeasy Mini Kit (QIAGEN) with a one-hour in-tube DNase digestion (QIAGEN) to remove possible DNA contamination according to the manufacturer’s instructions. Two biological replicates of each sample were prepared. The concentration of RNA was determined using a Spectrophotometer (ND-1000) (NanoDrop).

For quantitative real-time RT-PCR, melting curve analyses were performed after each reaction to ensure amplification specificity. Differences (n-fold) in transcripts were calculated using the relative comparison method, and amplification efficacies of each primer set were verified as described by Schmittgen et al. [29]. RNA levels were normalized using the housekeeping gene *tus* encoding DNA replication terminus site-binding protein as endogenous control [30]. Quantitative real-time RT-PCR (qRT-PCR) was performed with a Bio-Rad iQ5 iCycler detection system using iScript one-step RT-PCR kit with SYBR Green (Bio-Rad) according to the manufacture’s instruction [31].

### Statistical analysis

Pathogenicity and lethality scores, resistance and number of virulence-associated genes (VAGs) were treated as quantitative variables and described by mean ± standard deviation (SD). Data was analyzed using non-parametric tests due to asymmetry in their distributions, except for number of VAGs. For comparisons among ECOR groups, one-way ANOVA and the Kruskal-Wallis methods were used. The relationship between the presence of a gene and the pathogenicity score was analyzed using the Mann-Whitney test, by comparing the scores in isolates with and without this particular gene. All statistical analysis was carried out with the Statistical Package for the Social Sciences (IBM SPSS v.18.0) or WinPEPI v.11.18 (Abramson, J.H. WINPEPI updated: computer programs for epidemiologists, and their teaching potential. Epidemiologic Perspectives & Innovations 2011, 8:1). Statistical significance was accepted at *p*≤0.05.

## Results

### Antibiotic resistance among the APEC isolates

**Figure 1:**
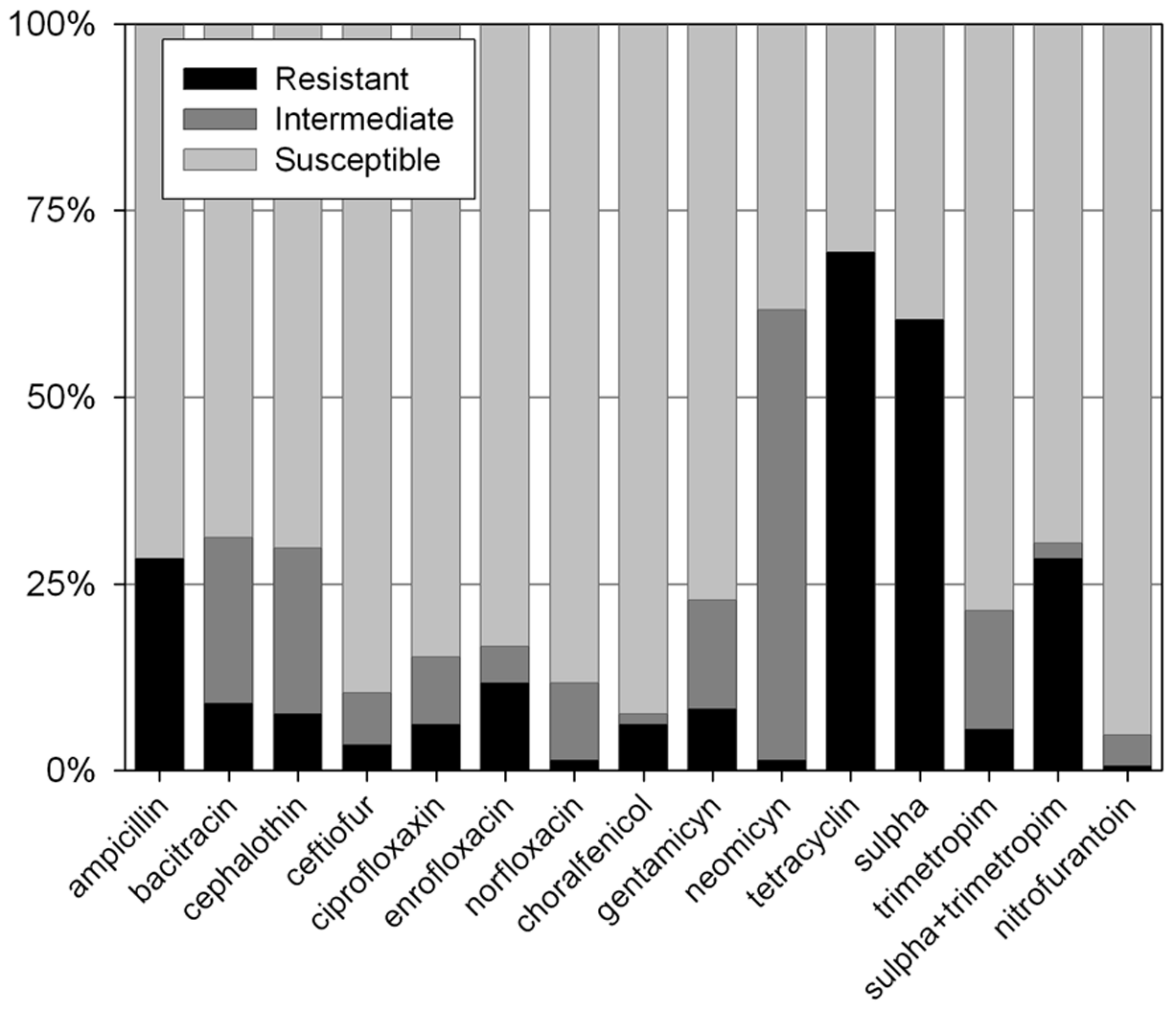
Antimicrobial susceptibility of cellulitis isolates. The susceptibility of 144 APEC isolates to 15 antimicrobials was tested individually using disc diffusion tests.

**Figure 2:**
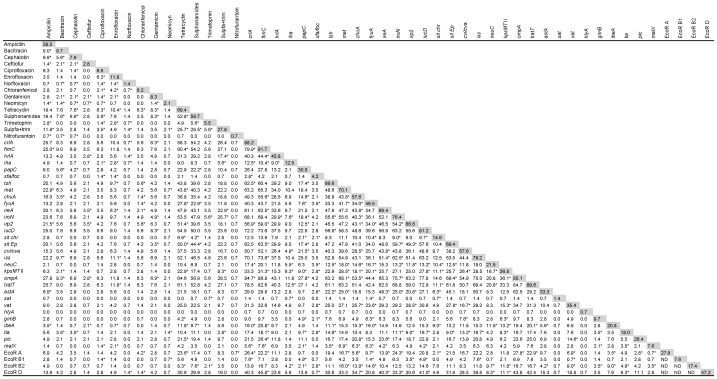
Association between resistance to 15 antimicrobials, presence of 31 VAGs^1^ and ECOR group among 144 APEC isolates. Numbers indicate the percentage of isolates that have both traits, while numbers in shadowed boxes indicate the percentage of isolates that have the corresponding trait; * *p*≤0.001 using χ^2^. ND, not determined.^1^ *afa* and *cnf* are not included, since they were not present in any strain, and *csgA* is not included, since it was present in all strains.

All 144 APEC isolates were tested for susceptibility to 15 antimicrobial agents that have, at some point, been commonly employed in the Brazilian poultry industry, either as growth promoters, to prevent infection and/or for treatment. It was found that the APEC isolates were susceptible to the majority of antimicrobials. The frequency of resistance to all the antimicrobials was less than 30% except for tetracycline and sulphonamides (with frequencies of 69.4% and 59.7% resistance, respectively) (Fig.1). For the exact resistance values, see the shadowed boxes in Figure 2.

With respect to the sites of antibiotic action, 29.2% of the isolates were resistant to at least one of the antimicrobials that act on the cell wall (ampicillin, bacitracin, cephalothin, ceftiofur); 11.8% were resistant to at least one of the antimicrobials that inhibit nucleic acid synthesis (ciprofloxacin, enrofloxacin, norfloxacin); 68.0% were resistant to at least one of the antimicrobials that block protein synthesis (chloramphenicol, gentamicin, neomycin, tetracycline; but the percentage was only 15.3% if tetracycline is excluded); and 59.7% were resistant to at least one of the antimicrobials that target folate synthesis (sulphonamides, trimethoprim, sulpha + trimethoprim; but only 29.2% if sulphonamides are excluded).

When we analyzed multi-resistance patterns, we observed that 18% of all isolates were susceptible to, or had intermediate resistance to, all antibiotics tested. Twenty per cent were resistant to at least one agent; 17% to 2; 19% to 3; and 11% to 4. Fifteen per cent were resistant to 5 or more, and one strain (PR133) was resistant to 10. It is noteworthy that there was no antimicrobial agent to which all 144 APEC strains were susceptible. Figure 2 shows the percentage of strains with resistance to given pairs of antimicrobials.

### Genotyping by multiplex polymerase chain reaction

The prevalence of virulence-associated genes (VAGs) among the APEC isolates is shown in Table 1. The factors *fimC, ompA, traT, csgA* and *crlA* were the most frequent VAGs. Overall, the isolates had an average of 15.2 VAGs.

Virulence factors related to adhesion, iron acquisition and serum resistance were present in all strains, with the exception of strain PR010 (median PS 9.1; Fig. S1), which did not contain any of the iron acquisition systems tested. ColV plasmid-associated genes (*iroN, iss, iucD, sitD, traT, tsh*) occurred in the majority of the isolates (from 89.6 to 66.6%), although the ColV-encoding genes *cvi/cva* were present in only 57.6% of the isolates (Table 1). The factors *afa/dra* and *cnf1/2* were not detected in any isolate, while *csgA* was detected in all the isolates. The VAGs harbored by each isolate are presented in Figure S1.

### Lethality and pathogenicity tests

Lethality and pathogenicity tests in day-old chicks were used to evaluate the virulence of the APEC isolates as described in the Materials and Methods section, and the results for each strain are presented in Figure S1. Most isolates were lethal: 105 (72.9%) of the isolates killed all 10 chicks within 7 days (LS  =  10); 14 isolates (9.7%) killed 9 chicks, 9 isolates (6.2%) killed 8 chicks, 5 isolates (3.5%) killed 7, 4 isolates (2.8%) killed 6, 2 isolates (1.4%) killed 5, 3 isolates (2.1%) killed 4, 1 isolate killed 3 chicks (0.7%), and 1 killed 1 chicken (0.7%). Overall, the isolates had a lethality score of 9.26. None of the chicks inoculated with BHI or MG1655 (the negative controls) died.

**Figure 3:**
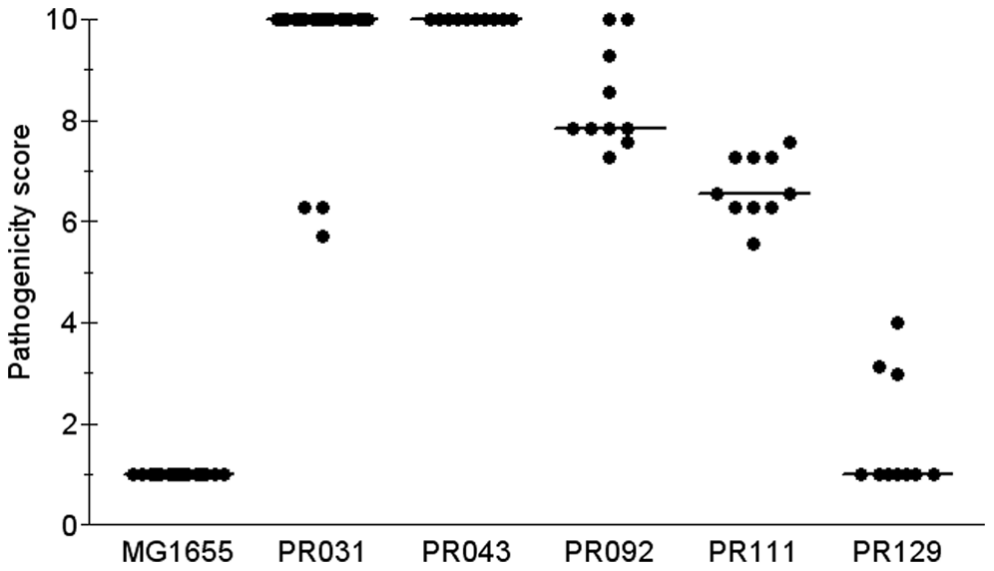
Pathogenicity scores for MG1655 and five cellulitis isolates. Ten one-day old chicks were infected with each isolate and observed for 7 days as described in the Materials and Methods section. Data points represent the PS for each chick, and horizontal bars represent the median PS for each isolate. Strain MG1655 was included as negative control.

In addition to the LS, we also determined the pathogenicity scores. The PS takes into account the clinical symptoms and how quickly the infection kills birds, in addition to how many chicks die within 7 days [22]. Thus, while an LS of 10 means that all 10 chicks died within seven days, a PS of 10 means that most of the 10 chicks died on the first day. Fifty-six isolates (38.1%) had a median PS  =  10; 36 (25%) had a 9.9 > median PS > 7.0; 42 (29.2%) had a 6.9 > median PS > 5.0; and 10 (6.9%) had a median PS lower than 5. Overall, the APEC isolates had a pathogenicity score of 8.01. Chicks inoculated with BHI displayed no signs of infection, while chicks inoculated with MG1655 displayed only small cellulitis lesions at the inoculation sites (Fig. 3). Figure 3 displays the data for a few isolates that illustrate different PS.

### PCR-based classification into ECOR phylogenetic groups

We performed phylogenetic typing of the APEC isolates using the Clermont method [24]. This technique uses the *chuA* and *YjaA* genes and *TspE4C2* fragment to allocate *E. coli* strains to phylogenetic groups A, B1, B2 and D. In previous studies, virulent extraintestinal strains were found to belong mainly to group B2 and, to a lesser extent, to group D, whereas most commensal strains belonged to group A [24], [25]. The distribution of our APEC isolates among the four phylogenetic groups is shown in Table 2. As determined by PCR, most of the strains (47.2%) belonged to group D.

**Table 2:** ECOR groups among the 144 cellulitis isolates (within parentheses, number of strains) and respective mean and standard deviation for antimicrobial resistance, number of VAGs, lethality scores and pathogenicity scores.

	ECOR groups (number of isolates)				
	A (40)	B1 (11)	B2 (25)	D (68)	*p* value
Resistance	2.9±1.9	2.7±2.2	1.8±1.8	2.5±2.1	0.144^3^
Resistance (-tetra,sulpha)^1^	1.4±1.6	1.4±1.9	0.9±1.2	1.2±1.6	0.441^3^
VAGs (34)	13.9±2.8 **a**	14.5±2.8 **a**	17.5±2.9 **b**	15.3±2.5 **a**	<0.001^4^
APEC VAGs (8)^2^	4.5±1.4	4.5±1.2	4.3±1.4	4.6±1.2	0.732^5^
Lethality score	9.6±0.9 **ab**	8.1±2.4 **b**	9.8±0.8 **a**	9.1±1.9 **b**	0.005^6^
Pathogenicity score	8.3±1.7 **ab**	7.3±2.7 **ab**	8.9±1.5 **a**	7.6±2.3 **b**	0.050^6^

1 Mean number of antimicrobials to which strains were resistant, excluding tetracycline and sulphonamides.

2 APEC VAGs: papC, tsh, irp2, iucD, cva/cvi, iss, astA and vat.

3 One-way Kruskal-Wallis.

4 One-way ANOVA; means indicated by the same letter did not differ using the SNK test (0.05 level).

5 One-way ANOVA.

6 One-way Kruskal-Wallis; means indicated by the same letter did not differ using the Kruskal-Wallis adjusted for multiple comparisons (0.05 level).

Table 2 also shows the mean number of antimicrobials to which the strains were resistant, and the mean number of VAGs and pathogenicity and lethality scores of strains according to their ECOR group. No statistical differences were observed among the phylogenetic groups in relation to resistance (*p* > 0.10). Isolates from group B2 possessed a significantly higher number of VAGs per strain, and the remaining groups did not differ in terms of the number of VAGs they possessed. Figure 2 shows the VAGs positively linked (*p*≤0.001) to ECOR groups A, B1, B2 and D. As for the lethality and pathogenicity scores, the B2 strains had on average the highest values, whereas the B1 and D groups had the lowest values.

In an attempt to find out what increases the virulence of APEC isolates in one-day-old chicks, we looked for significant associations between the presence of VAGs and pathogenicity scores (Table 3). We found that a higher PS was positively linked (*p*≤0.05) to the VAGs *kpsMTII*, *gimB* and *ibeA*, but negatively linked (*p*≤0.005) to *pic*.

**Table 3:** Relationship between APEC pathogenicity score (PS) and presence of different genes.

	Gene +		Gene -		
Gene^a^	Average PS	Number of isolates	Average PS	Number of isolates	*p* b
***crlA***	8.11	127	7.26	17	0.300
***fimC***	7.92	132	9.01	12	0.100
***hrlA***	7.93	66	8.08	78	0.377
***iha***	8.28	18	7.97	126	0.925
***papC***	8.37	44	7.86	100	0.264
***sfa/foc***	8.88	6	7.98	138	0.377
***tsh***	7.87	96	8.31	48	0.269
***mat***	8.09	101	7.82	43	0.559
***chuA***	7.96	83	8.09	61	0.756
***fyuA***	7.84	67	8.16	77	0.282
***ireA***	7.84	100	8.40	44	0.204
***iroN***	8.01	110	8.01	34	0.886
***irp2***	7.87	96	8.29	48	0.344
***iucD***	8.03	117	7.93	27	0.770
***sit chr***	8.67	21	7.90	123	0.078
***sit Ep***	8.08	100	7.86	44	0.362
***cvi/cva***	8.24	83	7.71	61	0.176
***iss***	8.03	114	7.94	30	0.790
***neuC***	7.69	31	8.10	113	0.663
***kpsMT*** **II**	8.61	53	7.66	91	0.004*
***ompA***	7.99	137	8.39	7	0.865
***tratT***	7.96	129	8.49	15	0.443
***astA***	7.89	48	8.08	96	0.864
***sat***	6.25	2	8.04	142	-
***vat***	7.84	51	8.11	93	0.475
***hlyA***	6.28	1	8.03	143	-
***gimB***	9.10	14	7.90	130	0.026*
***ibeA***	8.56	30	7.87	114	0.042*
***tia***	8.60	26	7.88	118	0.178
***pic***	7.11	38	8.34	106	0.006*
***malX***	8.09	11	8.01	133	0.844

a Genes that occurred in none (*afa/dra*, *cnf1/2)* or all (*csgA*) isolates are not listed.

b Exact *p* values for the Wilcoxon-Mann-Whitney test.

* *p*≤0.05.

### Expression of *kpsMTII*, *gimB*, *ibeA* and *pic*

**Figure 4:**
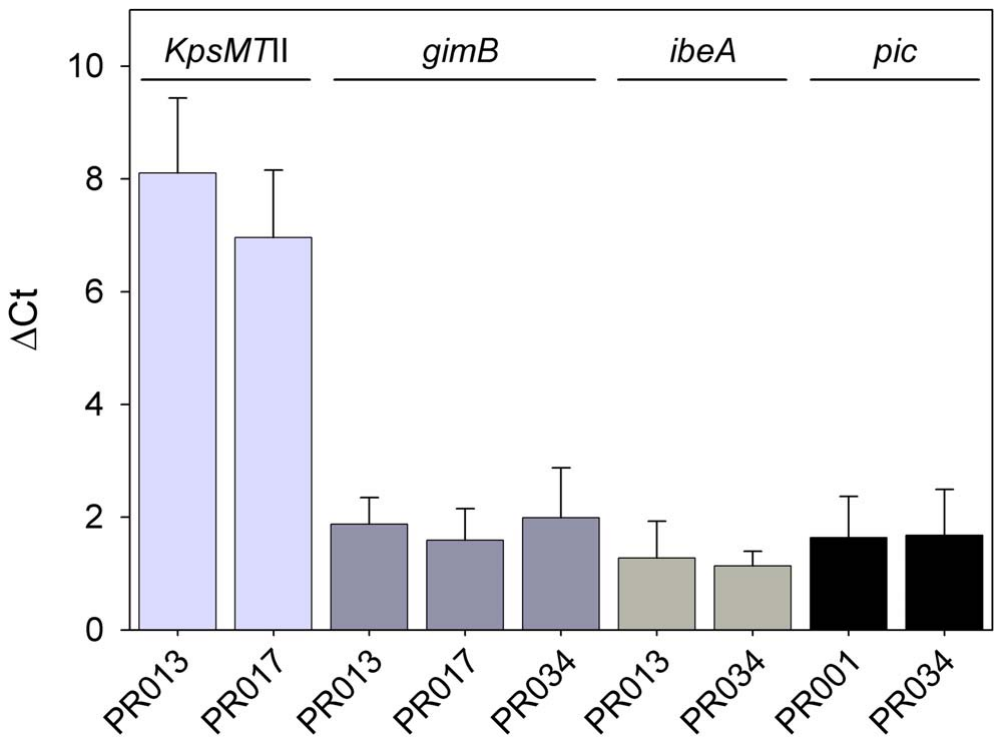
Expression of genes found to influence the PS. Real-time quantitative RT-PCR was used to analyze the expression of *kpsMTII*, *gimB*, *ibeA* and *pic* in PR001, PR013, PR017 and PR034. ▵ct expresses ct_mean_ subtracted of ct_mean_ of *tus* (housekeeping gene) of the respective isolate. Data represent the average ± SD of two experiments done in triplicates.

We quantified the expression of *kpsMTII*, *gimB*, *ibeA* and *pic* in four strains that contain different combinations of these VAGs, namely PR01 (*pic*), PR013 (*kpsMTII*, *gimB* and *ibeA*), PR017 (*kpsMTII* and *gimB*) and PR034 (*gimB*, *ibeA* and *pic*), using *tus* as a housekeeping gene. Figure 4 shows that all strains expressed these genes.

### Phylogenetic Analysis

We used the ARDRA method to evaluate genetic diversity among the 144 isolates. A similarity matrix was generated based on the presence or absence of restriction bands and strains were clustered accordingly. The ARDRA profiles of the strains are shown in Figure S2. We found 104 different ARDRA profiles among the 144 isolates; 82 of them had distinct band patterns, while the remaining 62 isolates fell into 22 groups each containing 2 to 6 strains with the same band pattern. The majority of isolates with similar band patterns came from different farms, and only 4 of the 22 groups contained strains collected from the same farm (column “Origin” in Fig. S2). It is noteworthy that strains with the same band pattern have different virulence genotypes and could belong to distinct ECOR groups.

From the dendrogram, eight major clusters could be grouped at a similarity level of 80% or more (Fig. S2, right), with the majority of isolates falling into clusters A and B.

## Discussion

Antimicrobial therapy is one of the primary control measures for reducing the morbidity and mortality caused by APEC infections. Since the indiscriminate use of antimicrobials leads to the selection of resistant isolates, they need to be used prudently in order to preserve their therapeutic usefulness in both animals and humans [32]. Among the 144 APEC strains isolated from cellulitis lesions in Brazil, the highest frequency of resistance was against tetracycline (69.4%), in agreement with the observations of others [33], [34], [35], [36], [37], [38], [39], [40]. This is not surprising since tetracycline has been used extensively for several decades, primarily as a feed additive in the poultry industry, and is the oldest antimicrobial on the market that was tested in this work. As a consequence, resistance to tetracycline has increased over the years.

Apart from tetracycline and sulphonamides, the frequency of resistance to the other antimicrobials was fairly low. Despite that, resistance to at least one antimicrobial was observed in almost all isolates (85.9%), and about half (54.6%) exhibited resistance to three or more antibiotics, as observed in previous work [35], [39], [40]. Frequencies of resistance to nitrofurantoin and sulpha + trimethoprim were much lower in the strains collected between 2006-2007 (this work) than in strains collected between 1998-2000 within the same Southern Brazilian region [2]. We expect that the increasing restrictions on the use of antimicrobials in the poultry industry will, in the near future, result in a considerably lower frequency of resistance of APEC strains to antimicrobial agents.

Our phylogenetic typing results showed that the APEC mainly belong to *E. coli* reference collection (ECOR) group D, in contrast to studies in the USA, China and Germany, in which most isolates were found to be in group A [17], [23], [41], [42], and in France where they were found to be in group B2 [43]. In all the cited studies, including ours, isolates belonging to group B1 were rare. Extraintestinal isolates from poultry seem, therefore, to be broadly distributed between groups A, B2 and D, but not group B1. It is important to note, however, that the multiplex-PCR method of Clermont et al. (2000) may sometimes classify strains actually belonging to group B1 as group A [24].

An APEC is defined as an *E. coli* isolated from an extraintestinal infection of birds. Since infections can be localized or systemic, and caused by more or less virulent strains, an APEC collection will include isolates with widely varying degrees of virulence. It can be argued that the higher the virulence of an APEC, the greater its potential to cause a systemic infection. Moreover, immunocompromised birds can be infected with less virulent APECs or even avirulent commensal *E. coli*. The genotyping of the 144 cellulitis isolates revealed that all of them harbor virulence factors related to adhesion, iron acquisition (with the exception of strain PR010) and serum resistance, which are characteristic of the APEC pathotype. Thus, as in previous reports [2], [4], [36], our results demonstrate that cellulitis APEC isolates are indistinguishable from septicemic APEC isolates on the basis of virulence factors. Yet the genotype does not guarantee that an extraintestinal *E. coli* is truly an APEC, so *in vivo* assays are necessary to confirm that the isolate does indeed cause infection.

Since it is not feasible to perform *in vivo* virulence tests on a large number of strains in 3- or 5-week-old chickens, large APEC collections are tested in one-day-old chicks [2], [14], [28], [43]. To analyze the virulence of our strains, we used lethality tests (LS) on one-day-old chicks. The majority of our strains were lethal to these chicks within 7 days, strongly suggesting that they were virulent APEC.

To better analyze the virulence of a given strain, we combined an analysis of organ lesions with how quickly the infection killed the chicks, to provide a pathogenicity score (PS), which is more likely to detect differences in virulence among APEC strains than the lethality score. Mortality and organ lesions in one-day-old chicks have previously been used to classify APEC strains as having high, intermediate or low pathogenicity [16], [44]. On the basis of their classification, the majority of our strains were found to be highly pathogenic; only ten had a PS lower than 5. Our phylogenetic analysis using ARDRA failed to identify a prevalent APEC clone. Instead, the population of cellulitis isolates proved to be diverse, with few strains belonging to the same clone.

We then analyzed which genes influenced the PS; we found that the presence of genes for invasins *ibeA* and *gimB* and for group II capsule *KpsMT*II increased virulence, while the presence of *pic* decreased virulence. The expression of these genes in BHI was confirmed by quantitative RT-PCR in four isolates. In agreement with these findings, it has been observed that APEC strains MT78 and IMT5155 caused a systemic infection when inoculated intratracheally into 5-week-old chickens, while UEL17 remained restricted to the lungs [8]. The main differences in the virulence genotypes among the three strains are the absence of *ibeA*, *gimB*, *neuC* and *KpsMT*II, and presence of *pic* in UEL17 [8]. Genes *ibeA* and *KpsMT*II have been associated with APEC virulence [45], [46]. Although *ibeA*, *gimB* and *KpsMT*II were not found to be present in the majority of APEC strains (Table 1) [17], [23], [45], [47] and cannot, therefore, be considered defining traits of APEC, they may be “significant but minority traits” in increasing APEC virulence [47]. Since *ibeA*, *gimB* and *KpsMT*II are characteristic of NMEC [23], [48], we may conclude that the genes that render an APEC more similar to NMEC increase virulence, and the zoonotic risk. Interestingly, Mora et al. [49] observed that the extraintestinal *E. coli* clonal group O25b:K1:H4-ST131 harboring *ibeA* and *KpsMT*II has recently emerged among APEC isolates.

The gene *pic*, like *tsh*, encodes a serine protease autotransporter protein, and was included in the screening of VAGs in APEC isolates [15], [23] because it had been implicated in UPEC virulence [50]. However, according to our results, the presence of *pic* was associated with decreased virulence of APEC in day-old chicks (Table 3). The construction of a *pic* mutant would help to elucidate its role in APEC virulence.

In summary, in this work, we genotyped and performed virulence tests *in vivo* on the largest number of APEC isolates from severe lesions of cellulitis described so far. In addition, our data provide a comprehensive overview of the susceptibility of cellulitis isolates currently found in south Brazil to antimicrobials and their phylogenetic status.

## Supporting Information

Figure S1 **Characterization of 144 APEC isolates.** Columns from left to right: S*train*, isolate designation; *Origin*, source of the isolate; *Resistance*, number of antimicrobials to which the isolate was resistant; the subsequent columns depict the PCR results for all VAGs tested, with presence indicated in black and absence indicated in white (except for *afa* and *cnf* ½, which were absent from all isolates); *no. VAGs*, total number of VAGs in each isolate; *LS*, lethality score; *PS median (range)*, median pathogenicity score (range); *PS mean ± SD*, mean pathogenicity score ± standard deviation; *ECOR*, ECOR phylogenetic group; *Cluster*, strains with 80% similarity were grouped into 8 genotypic clusterings (A to H).
(PDF)

Figure S2 **ARDRA profile of 144 APEC isolates.** The ARDRA dendrogram was constructed by UPGMA based upon enzyme restriction digestion of amplified 16-23S DNA intergenic spacer regions. The column S*train* shows isolate designation; the column *Origin*, source of isolate, with 1 to 65 designating each of the 65 farms from which the isolates were collected. *Cluster* designates the 8 genotypic clusters (A to H) into which strains with 80% similarity were grouped. *E. coli* ATCC25922 was analyzed as a reference strain.
(TIF)

## References

[1] Dho-Moulin M, Fairbrother JM (1999) Avian pathogenic *Escherichia coli* (APEC). Veterinary Research 30: 299–316. 10367360

[2] Brito BG, Gaziri LCJ, Vidotto MC (2003) Virulence factors and clonal relationships among *Escherichia coli* strains isolated from broiler chickens with cellulitis. Infection and Immunity 71: 4175–4177. 12819112 10.1128/IAI.71.7.4175-4177.2003 PMC162012

[3] Jeffrey JS, Nolan LK, Tonooka KH, Wolfe S, Giddings CW, et al (2002) Virulence factors of *Escherichia coli* from cellulitis or colisepticemia lesions in chickens. Avian Diseases 46: 48–52. 11922349 10.1637/0005-2086(2002)046[0048:VFOECF]2.0.CO;2

[4] Ngeleka M, Kwaga JKP, White DG, Whittam TS, Riddell C, et al (1996) *Escherichia coli* cellulitis in broiler chickens: Clonal relationships among strains and analysis of virulence-associated factors of isolates from diseased birds. Infection and Immunity 64: 3118–3126. 8757842 10.1128/iai.64.8.3118-3126.1996 PMC174196

[5] Norton RA, Bilgili SF, McMurtrey BC (1997) A reproducible model for the induction of avian cellulitis in broiler chickens. Avian Diseases 41: 422–428. 9201408

[6] Fallavena LCB, Moraes HLS, Salle CTP, da Silva AB, Vargas RS, et al (2000) Diagnosis of skin lesions in condemned or downgraded broiler carcasses - a microscopic and macroscopic study. Avian Pathology 29: 557–562. 19184851 10.1080/03079450020016797

[7] UBABEF (2011) Relatório Anual 2010/2011. Available: www.ubabef.com.br. Accessed 2013 March 20.

[8] Horn F, Corrêa AMR, Barbieri NL, Glodde S, Weyrauch K-D, et al (2012) Infections with avian pathogenic and fecal *Escherichia coli* strains display similar lung histopathology and macrophage apoptosis. PLoS ONE 7: e41031. 22848424 10.1371/journal.pone.0041031 PMC3405075

[9] Dziva F, Stevens MP (2008) Colibacillosis in poultry: unravelling the molecular basis of virulence of avian pathogenic *Escherichia coli* in their natural hosts. Avian Pathology 37: 355–366. 18622850 10.1080/03079450802216652

[10] Johnson TJ, Johnson SJ, Nolan LK (2006) Complete DNA sequence of a ColBM plasmid from avian pathogenic Escherichia coli suggests that it evolved from closely related ColV virulence plasmids. Journal of Bacteriology 188: 5975–5983. 16885466 10.1128/JB.00204-06 PMC1540072

[11] Johnson TJ, Siek KE, Johnson SJ, Nolan LK (2006) DNA sequence of a ColV plasmid and prevalence of selected plasmid-encoded virulence genes among avian *Escherichia coli* strains. Journal of Bacteriology 188: 745–758. 16385064 10.1128/JB.188.2.745-758.2006 PMC1347294

[12] Mellata M, Touchman JW, Curtiss R III (2009) Full Sequence and Comparative Analysis of the Plasmid pAPEC-1 of Avian Pathogenic E-coli chi 7122 (O78:K80:H9). PLoS ONE 4: e4232 doi: 4210.1371/journal.pone.0004232 19156210 10.1371/journal.pone.0004232 PMC2626276

[13] Mellata M, Ameiss K, Mo H, Curtiss R III (2010) Characterization of the contribution to virulence of three large plasmids of avian pathogenic *Escherichia coli* chi 7122 (O78:K80:H9). Infection and Immunity 78: 1528–1541. 20086082 10.1128/IAI.00981-09 PMC2849417

[14] Schouler C, Schaeffer B, Brée A, Mora A, Dahbi G, et al (2012) Diagnostic strategy for identifying avian pathogenic *Escherichia coli* based on four patterns of virulence genes. Journal of Clinical Microbiology 50: 1673–1678. 22378905 10.1128/JCM.05057-11 PMC3347144

[15] Ewers C, Antao E-M, Diehl I, Philipp H-C, Wieler LH (2009) Intestine and environment of the chicken as reservoirs for extraintestinal pathogenic *Escherichia coli* strains with zoonotic potential. Applied and Environmental Microbiology 75: 184–192. 18997030 10.1128/AEM.01324-08 PMC2612213

[16] Rosenberger JK, Fries PA, Cloud SS, Wilson RA (1985) *In vitro* and *in vivo* characterizaion of avian *Escherichia coli*. 2. Factors associated with pathogenicity. Avian Diseases 29: 1094–1107. 3914272

[17] Johnson TJ, Wannemuehler Y, Doetkott C, Johnson SJ, Rosenberger SC, et al (2008) Identification of minimal predictors of avian pathogenic *Escherichia coli* virulence for use as a rapid diagnostic tool. Journal of Clinical Microbiology 46: 3987–3996. 18842938 10.1128/JCM.00816-08 PMC2593276

[18] CLSI (2009) Performance Standards for Antimicrobial Susceptibility Testing; Aproved Standard- Tenth Edition M02-A10. 940 West Valley Road, Suite 1400, Wayne, Pennsylvania 19087-1898 USA: Clinical and Laboratory Standards Institute.

[19] CLSI (2008) Performance Standards for Antimicrobial disk and dilution susceptibility tests for bacteria isolated from animals; Aproved Standard- Third Edition. CLSI document M31- A3. Pennsylvania: Clinical and Laboratory Standards Institute

[20] Up JP (2003) Notice of Approval of New Animal Drug Application; Ceftiofur - Supplement to NADA 140-890 In: New Animal Drug Evaluation CfVM, editor.

[21] Sayah RS, Kaneene JB, Johnson Y, Miller R (2005) Patterns of Antimicrobial Resistance Observed in *Escherichia coli* Isolates Obtained from Domestic- and Wild-Animal Fecal Samples, Human Septage, and Surface Water. Applied and Environmental Microbiology 71: 1394–1404. 15746342 10.1128/AEM.71.3.1394-1404.2005 PMC1065171

[22] Barbieri NL, Tejkowski TM, Oliveira AL, Brito BG, Horn F (2012) Characterization of extra-intestinal *Escherichia coli* isolated from a peacock (*Pavo cristatus*) with colisepticemia. Avian Diseases 56: 436–440. 22856209 10.1637/9921-090811-Case.1

[23] Ewers C, Li GW, Wilking H, Kiessling S, Alt K, et al (2007) Avian pathogenic, uropathogenic, and newborn meningitis-causing *Escherichia coli*: How closely related are they? International Journal of Medical Microbiology 297: 163–176. 17374506 10.1016/j.ijmm.2007.01.003

[24] Clermont O, Bonacorsi S, Bingen E (2000) Rapid and simple determination of the *Escherichia coli* phylogenetic group. Applied and Environmental Microbiology 66: 4555–4558. 11010916 10.1128/aem.66.10.4555-4558.2000 PMC92342

[25] Herzer PJ, Inouye S, Inouye M, Whittam TS (1990) Phylogenetic distribution of branched RNA-linked multicopy single-stranded-DNA among natural isolates of *Escherichia coli* . Journal of Bacteriology 172: 6175–6181. 1699928 10.1128/jb.172.11.6175-6181.1990 PMC526797

[26] Garcia-Martinez J, Martinez-Murcia AJ, Rodriguez-Valera F, Zorraquino A (1996) Molecular evidence supporting the existence of two major groups in uropathogenic *Escherichia coli* . FEMS Immunology and Medical Microbiology 14: 231–244. 8856322 10.1111/j.1574-695X.1996.tb00291.x

[27] Anton AI, Martinez-Murcia AJ, Rodriguez-Valera F (1998) Sequence diversity in the 16S-23S intergenic spacer region (ISR) of the rRNA operons in representatives of the *Escherichia coli* ECOR collection. Journal of Molecular Evolution 47: 62–72. 9664697 10.1007/pl00006363

[28] Vidotto MC, Muller EE, de Freitas JC, Alfieri AA, Guimaraes IG, et al (1990) Virulence factors of avian *Escherichia coli* . Avian Diseases 34: 531–538. 2241678

[29] Schmittgen TD, Zakrajsek BA, Mills AG, Gorn V, Singer MJ, et al (2000) Quantitative reverse transcription-polymerase chain reaction to study mRNA decay: Comparison of endpoint and real-time methods. Analytical Biochemistry 285: 194–204. 11017702 10.1006/abio.2000.4753

[30] Skyberg JA, Johnson TJ, Nolan LK (2008) Mutational and transcriptional analyses of an avian pathogenic *Escherichia coli* CoIV plasmid. BMC Microbiology 8: 24. 18230176 10.1186/1471-2180-8-24 PMC2270849

[31] Li G, Tivendale KA, Liu P, Feng Y, Wannemuehler Y, et al (2011) Transcriptome analysis of avian pathogenic *Escherichia coli* O1 in chicken serum reveals adaptive responses to systemic infection. Infection and Immunity 79: 1951–1960. 21357721 10.1128/IAI.01230-10 PMC3088125

[32] Gyles C (2008) Antimicrobial resistance in selected bacteria from poultry. Animal Health Research Reviews 9: 149–158. 19102788 10.1017/S1466252308001552

[33] Blanco JE, Blanco M, Mora A, Blanco J (1997) Prevalence of bacterial resistance to quinolones and other antimicrobials among avian *Escherichia coli* strains isolated from septicemic and healthy chickens in Spain. Journal of Clinical Microbiology 35: 2184–2185. 9230413 10.1128/jcm.35.8.2184-2185.1997 PMC229934

[34] Cormican M, Buckley V, Corbett-Feeney G, Sheridan F (2001) Antimicrobial resistance in *Escherichia coli* isolates from turkeys and hens in Ireland. Journal of Antimicrobial Chemotherapy 48: 587–588. 11581244 10.1093/jac/48.4.587

[35] Ozawa M, Harada K, Kojima A, Asai T, Sameshima T (2008) Antimicrobial susceptibilities, serogroups, and molecular characterization of avian pathogenic *Escherichia coli* isolates in Japan. Avian Diseases 52: 392–397. 18939625 10.1637/8193-120907-Reg

[36] Peighambari SM, Vaillancourt JP, Wilson RA, Gyles CL (1995) Characteristics of *Escherichia coli* isolates from avian cellulitis. Avian Diseases 39: 116–124. 7794170

[37] Schroeder CM, Meng JH, Zhao SH, DebRoy C, Torcolini J, et al (2002) Antimicrobial resistance of *Escherichia coli* O26, O103, O111, O128, and O145 from animals and humans. Emerging Infectious Diseases 8: 1409–1414. 12498656 10.3201/eid0812.020770 PMC3369591

[38] Wooley RE, Spears KR, Brown J, Nolan LK, Fletcher OJ (1992) Relationship of complement resistance and selected virulence factors in pathogenic avian *Escherichia coli* . Avian Diseases 36: 679–684. 1417597

[39] Yang HC, Chen S, White DG, Zhao SH, McDermott P, et al (2004) Characterization of multiple-antimicrobial-resistant *Escherichia coli* isolates from diseased chickens and swine in China. Journal of Clinical Microbiology 42: 3483–3489. 15297487 10.1128/JCM.42.8.3483-3489.2004 PMC497637

[40] Zhao SH, Maurer JJ, Hubert S, De Villena JF, McDermott PF, et al (2005) Antimicrobial susceptibility and molecular characterization of avian pathogenic *Escherichia coli* isolates. Veterinary Microbiology 107: 215–224. 15863280 10.1016/j.vetmic.2005.01.021

[41] Rodriguez-Siek KE, Giddings CW, Doetkott C, Johnson TJ, Fakhr MK, et al (2005) Comparison of *Escherichia coli* isolates implicated in human urinary tract infection and avian colibacillosis. Microbiology-Sgm 151: 2097–2110. 10.1099/mic.0.27499-0 15942016

[42] Zhao L, Gao S, Huan H, Xu X, Zhu X, et al (2009) Comparison of virulence factors and expression of specific genes between uropathogenic *Escherichia coli* and avian pathogenic *E. coli* in a murine urinary tract infection model and a chicken challenge model. Microbiology-Sgm 155: 1634–1644. 10.1099/mic.0.024869-0 19372154

[43] Moulin-Schouleur M, Schouler C, Tailliez P, Kao M-R, Bree A, et al (2006) Common virulence factors and genetic relationships between O18 : K1 : H7 *Escherichia coli* isolates of human and avian origin. Journal of Clinical Microbiology 44: 3484–3492. 17021071 10.1128/JCM.00548-06 PMC1594794

[44] Chen X, Yin J, Huan H, Gao S, Jiao X, et al (2012) Serogroups, pathogenicity and virulence-associated genes of avian *Escherichia coli* isolates collected in China. African Journal of Microbiology Research 6: 1001–1007.

[45] Germon P, Chen YH, He L, Blanco JE, Bree A, et al (2005) *ibeA*, a virulence factor of avian pathogenic *Escherichia coli* . Microbiology 151: 1179–1186. 15817785 10.1099/mic.0.27809-0

[46] Li GW, Laturnus C, Ewers C, Wieler LH (2005) Identification of genes required for avian *Escherichia coli* septicemia by signature-tagged mutagenesis. Infection and Immunity 73: 2818–2827. 15845486 10.1128/IAI.73.5.2818-2827.2005 PMC1087346

[47] Rodriguez-Siek KE, Giddings CW, Doetkott C, Johnson TJ, Nolan LK (2005) Characterizing the APEC pathotype. Veterinary Research 36: 241–256. 15720976 10.1051/vetres:2004057

[48] Bonacorsi S, Clermont O, Houdouin W, Cordevant C, Brahimi N, et al (2003) Molecular analysis and experimental virulence of french and North American *Escherichia coli* neonatal meningitis isolates: Identification of a new virulent clone. Journal of Infectious Diseases 187: 1895–1906. 12792866 10.1086/375347

[49] Mora A, Herrera A, Mamani R, Lopez C, Pilar Alonso M, et al (2010) Recent Emergence of Clonal Group O25b:K1:H4-B2-ST131 ibeA Strains among Escherichia coli Poultry Isolates, Including CTX-M-9-Producing Strains, and Comparison with Clinical Human Isolates. Applied and Environmental Microbiology 76: 6991–6997. 20817805 10.1128/AEM.01112-10 PMC2976257

[50] Heimer SR, Rasko DA, Lockatell CV, Johnson DE, Mobley HLT (2004) Autotransporter genes *pic* and *tsh* are associated with *Escherichia coli* strains that cause acute pyelonephritis and are expressed during urinary tract infection. Infection and Immunity 72: 593–597. 14688142 10.1128/IAI.72.1.593-597.2004 PMC343984

