# Supplementary material for: Genotypes and Pathogenicity of Cellulitis Isolates Reveal Traits That Modulate APEC Virulence
Source: PLoS One. 2013 Aug 19;8(8):e72322. doi: 10.1371/journal.pone.0072322 (PMC3747128; doi:10.1371/journal.pone.0072322)
Supplement: Figure S1 — Characterization of 144 APEC isolates. Columns from left to right: Strain, isolate designation; Origin, source of the isolate; Resistance, number of antimicrobials to which the isolate was resistant; the subsequent columns depict the PCR results for all VAGs tested, with presence indicated in black and absence indicated in white (except for afa and cnf ½, which were absent from all isolates); no. VAGs, total number of VAGs in each isolate; LS, lethality score; PS median (range), median pathogenicity score (range); PS mean ± SD, mean pathogenicity score ± standard deviation; ECOR, ECOR phylogenetic group; Cluster, strains with 80% similarity were grouped into 8 genotypic clusterings (A to H). (PDF) [file pone.0072322.s001.pdf]

Strain	Origin	Resistance	*csgA*	*crlA*	*fimC*	*hrlA*	*iha*	*papC*	*sfa/foc*	*tsh*	*mat*	*chuA*	*fyuA*	*ireA*	*iroN*	*irp2*	*iucD*	*sit chr*	*sit Ep*	*cvi/cva*	*iss*	*neuC*	*kpsMTII*	*ompA*	*traT*	*astA*	*sat*	*vat*	*hlyA*	*gimB*	*ibeA*	*tia*	*pic*	*malX*	VAGs	LS	PS median (range)	PS mean ± SD	ECOR	**Cluster**
PR025	63	2																																	11	10	8.6 (5.3-10)	8.1±2.1	D	
PR146	22	3																																	15	10	8.6 (3.9-10)	8.0±2.3	A	
PR141	42	2																																	12	10	7.9 (3.9-10)	7.8±2.0	A	
PR002	2	1																																	12	10	10 (5.6-10)	8.3±2.2	D	
PR028	64	0																																	14	10	10 (5.3-10)	8.8±2.0	D	
PR011	2	2																																	15	10	10 (10-10)	10±0	D	
PR006	2	0																																	16	7	7.3 (0-9.3)	5.4±3.6	A	
PR126	44	4																																	15	9	5.7 (2.0-8.6)	5.9±2.4	D	
PR133	38	10																																	19	10	7.4 (6.3-10)	8.1±1.7	D	
PR046	53	1																																	18	10	10 (5.3-10)	8.9±1.9	B2	
PR095	38	4																																	13	10	10 (6.3-10)	9.0±1.7	D	
PR007	2	3																																	10	8	7.6 (1.0-8.6)	6.9±2.4	B1	
PR065	3	5																																	10	10	10 (6.3-10)	9.6±1.2	A	
PR027	64	0																																	16	10	10 (6.3-10)	9.6±1.2	D	
PR049	46	1																																	21	10	7.3 (6.3-10)	7.8±1.5	D	
PR062	9	0																																	15	10	10 (6.3-10)	8.6±1.8	D	
PR036	57	2																																	14	10	10 (5.3-10)	9.3±1.6	D	
PR044	61	1																																	18	10	10 (5.3-10)	9.2±1.8	D	
PR080	14	2																																	16	10	8.3(3.9-10)	8.0±1.7	D	**A**
PR098	24	2																																	16	8	6.4 (1.0-10)	6.3±2.8	B1	
PR069	41	3																																	10	10	6.3 (5.3-10)	6.9±1.4	A	
PR066	42	5																																	12	10	10 (6.3-10)	8.8±1.6	A	
PR105	20	9																																	15	10	6.2 (4.1-9.3)	6.7±1.7	A	
PR143	32	3																																	16	10	10 (6.3-10)	9.4±1.4	D	
PR047	47	1																																	10	10	9.6 (6.3-10)	8.9±1.4	D	
PR048	60	1																																	12	5	5.1 (1.0-8.3)	4.6±3.1	B1	
PR014	2	1																																	14	6	3.9 (1.0-8.6)	3.3±2.3	B1	
PR103	20	3																																	18	10	6.4 (3.1-8.3)	6.6±1.5	A	
PR076	19	1																																	14	10	7.3 (6.3-10)	7.6±1.8	D	
PR034	51	2																																	19	10	10 (7.3-10)	9.5±1.1	B1	
PR108	34	5																																	14	10	10 (6.3-10)	9.5±1.2	A	
PR010	2	0																																	10	10	9.1 (6.3-10)	8.4±1.7	A	
PR023	48	1																																	16	10	8.6 (1.0-10)	7.4±2.6	D	
PR054	45	1																																	14	9	6.4 (4.0-10)	7.5±2.1	D	
PR052	45	0																																	15	5	6.3 (1.0-8.6)	5.0±3.4	D	
PR073	43	5																																	12	9	9.6 (1.0-10)	8.2±3.0	A	
PR100	30	3																																	15	9	5.9 (1.0-7.9)	5.7±2.1	A	
PR074	8	3																																	13	10	8.1 (4.1-10)	7.9±2.2	A	
PR110	8	3																																	16	10	6.9 (3.9-9.3)	7.0±1.6	B2	
PR136	8	2																																	12	10	8.8 (6.3-10)	8.3±1.8	A	
PR013	2	0																																	24	10	10 (5.3-10)	8.6±1.9	B2	
PR018	25	5																																	16	10	7.3 (5.6-10)	7.7±1.7	A	
Strain	Origin	Resistance	*csgA*	*crlA*	*fimC*	*hrlA*	*iha*	*papC*	*sfa/foc*	*tsh*	*mat*	*chuA*	*fyuA*	*ireA*	*iroN*	*irp2*	*iucD*	*sit chr*	*sit Ep*	*cvi/cva*	*iss*	*neuC*	*kpsMTII*	*ompA*	*traT*	*astA*	*sat*	*vat*	*hlyA*	*gimB*	*ibeA*	*tia*	*pic*	*malX*	VAGs	LS	PS median (range)	PS mean ± SD	ECOR	**Cluster**
PR001	2	2																																	16	8	6.1 (1.0-8.3)	5.2±2.6	D	
PR033	51	2																																	15	10	7.1 (4.9-10)	7.3±1.8	B1	
PR042	56	0																																	16	10	7.3 (6.3-10)	8.1±1.5	B2	
PR085	12	3																																	19	10	10 (5.3-10)	8.7±2.1	D	
PR148	21	4																																	14	10	10 (10-10)	10±0	D	
PR117	21	4																																	17	10	10 (9.3-10)	9.6±1.2	A	
PR024	63	2																																	13	4	2.5 (1.0-10)	4.3±3.7	D	
PR032	51	3																																	13	9	6.9 (1.0-8.3)	6.0±2.1	D	
PR031	51	0																																	16	10	10 (10-10)	10±0	A	
PR092	28	5																																	13	10	7.9 (7.3-10)	8.4±1.0	B2	
PR097	23	7																																	12	8	6.2 (2.0-7.6)	5.9±1.6	D	
PR106	17	7																																	17	10	10 (7.3-10)	9.7±0.9	D	
PR114	36	3																																	19	10	9.6 (7.6-10)	9.1±1.0	A	
PR144	29	3																																	14	9	6.9 (1.0-10)	6.6±2.8	D	
PR145	13	7																																	12	10	10 (4.6-10)	8.5±2.0	D	
PR096	44	4																																	17	10	6.3 (5.4-10)	6.8±1.7	D	
PR134	62	3																																	17	10	5.9 (4.9-10)	6.7±1.9	D	
PR124	22	3																																	16	10	5.6 (3.9-6.3)	5.3±0.9	D	
PR125	40	5																																	16	10	6.3 (5.9-10)	6.7±1.8	B2	
PR129	39	1																																	11	1	1 (1.0-4.0)	1.7±1.2	D	
PR030	54	7																																	14	10	10 (7.3-10)	9.7±0.9	B1	
PR147	17	4																																	15	10	6.8 (5.6-10)	7.5±1.8	D	
PR089	26	1																																	9	9	5.9 (4.0-9.3)	6.4±1.8	A	
PR022	48	1																																	18	9	4.7 (1.0-8.3)	5.5±2.3	D	
PR070	62	4																																	16	10	10 (5.3-10)	8.8±1.8	B2	
PR071	31	2																																	17	10	9.3 (5.3-10)	8.5±1.9	B2	
PR077	34	4																																	14	10	6.3 (5.3-10)	6.9±1.8	A	
PR093	13	6																																	15	10	9.6 (5.3-10)	8.4±1.9	D	
PR099	41	4																																	17	10	10 (5.3-10)	9.5±1.5	D	
PR068	9	0																																	13	10	10 (6.3-10)	8.7±1.7	D	
PR035	52	1																																	13	10	10 (5.3-10)	8.7±2.1	B2	
PR116	62	3																																	16	10	8.3 (5.6-10)	8.3±1.7	D	
PR119	22	2																																	15	10	6.8 (5.6-10)	7.2±1.6	D	
PR120	7	1																																	19	3	2.0 (1.0-5.0)	2.4±1.5	B1	
PR121	22	2																																	18	6	5.6 (1.0-7.3)	4.3±2.9	D	
PR094	27	3																																	18	10	8.6 (5.3-10)	8.0±2.1	D	
PR112	38	5																																	17	7	4.9 (1.0-9.3)	5.3±2.4	A	
PR037	58	2																																	17	10	10 (6.6-10)	9.7±1.1	D	**B**
PR107	33	5																																	15	10	6.3 (3.9-10)	6.7±1.9	D	
PR109	18	6																																	19	9	5.93 (3.9-10)	6.0±1.2	D	
PR058	26	4																																	15	7	5.6 (1.0-9.3)	5.0±3.1	D	
PR061	4	2																																	14	9	10 (1.0-10)	8.0±3.0	A	
PR130	26	3																																	14	9	6.4 (1.0-9.3)	6.1±2.2	A	
PR016	2	1																																	10	9	7.6 (5.0-10)	7.5±1.5	D	
PR084	24	1																																	16	7	5.9 (1.0-8.3)	5.1±3.0	D	
PR087	20	2																																	16	8	5.4 (1.0-9.3)	5.3±2.7	D	
PR086	41	5																																	12	10	8.2 (6.6-10)	8.4±1.3	A	
PR055	45	1																																	13	10	10 (4.6-10)	9.1±1.9	D	
PR078	34	3																																	17	10	10 (10-10)	10±0	A	
PR079	35	2																																	12	10	10 (6.3-10)	9.5±1.2	B1	
PR081	21	3																																	14	10	10 (9.3-10)	9.9±0.2	A	
PR082	34	3																																	15	10	9.6 (6.3-10)	8.6±1.6	A	
PR026	65	4																																	17	6	5.8 (1.0-8.3)	4.4±3.0	B2	
PR113	13	5																																	20	10	6.1 (3.9-10)	6.9±2.4	D	
PR127	32	4																																	17	10	6.9 (4.9-10)	7.7±2.1	D	
PR131	32	1																																	17	9	3.9 (1.0-5.6)	3.9±1.4	D	
PR021	48	3																																	13	9	9.3 (1.0-10)	7.8±3.0	A	
PR053	45	3																																	16	10	10 (6.3-10)	9.1±1.6	D	
PR122	28	2																																	15	4	2.5 (1.0-8.3)	3.3±2.6	D	
PR051	45	2																																	11	8	7.3 (1.0-10)	6.5±3.2	D	
PR128	31	1																																	22	10	10 (10-10)	10±0	A	
PR009	2	0																																	15	10	6.3 (5.3-10)	7.7±2.0	D	
PR041	55	0																																	18	10	10 (5.3-10)	9.1±2.0	D	
PR059	5	1																																	15	10	10 (6.3-10)	9.6±1.2	D	
PR090	37	0																																	20	10	10 (5.3-10)	8.7±1.8	B2	
PR104	5	1																																	20	10	10 (5.3-10)	9.1±1.6	B2	
PR135	40	2																																	16	10	10 (7.3-10)	9.5±1.1	B2	
PR057	58	0																																	19	10	10 (5.3-10)	8.4±2.1	B2	
PR111	37	2																																	20	10	6.6 (5.6-7.6)	6.7±0.6	B2	
PR132	21	1																																	17	10	10 (10-10)	10±0	A	
PR050	45	0																																	12	10	10 (10-10)	10±0	A	
PR056	58	0																																	19	10	6.3 (4.6-8.6)	6.3±1.5	B2	
PR072	23	4																																	17	10	10 (4.1-10)	8.7±2.3	B2	
PR139	6	2																																	12	10	10 (4.6-10)	9.0±1.9	A	
PR075	40	1																																	18	10	10 (5.3-10)	8.8±2.0	B2	
PR123	41	5																																	15	10	10 (5.3-10)	9.2±1.8	B2	
PR017	2	0																																	22	10	9.3 (7.6-10)	9.1±1.0	B2	
PR140	32	2																																	12	10	6.9 (3.9-10)	7.3±2.1	A	
Strain	Origin	Resistance	*csgA*	*crlA*	*fimC*	*hrlA*	*iha*	*papC*	*sfa/foc*	*tsh*	*mat*	*chuA*	*fyuA*	*ireA*	*iroN*	*irp2*	*iucD*	*sit chr*	*sit Ep*	*cvi/cva*	*iss*	*neuC*	*kpsMTII*	*ompA*	*traT*	*astA*	*sat*	*vat*	*hlyA*	*gimB*	*ibeA*	*tia*	*pic*	*malX*	VAGs	LS	PS median (range)	PS mean ± SD	ECOR	**Cluster**
PR012	2	0																																	15	10	6.9 (5.9-10)	7.3±1.5	A	
PR088	6	1																																	13	10	10 (5.6-10)	8.5±1.9	A	
PR083	41	4																																	18	10	10 (6.3-10)	9.6±1.2	D	C
PR138	16	1																																	12	10	7.4 (6.3-10)	7.9±1.6	B2	
PR101	34	0																																	16	8	6.4 (1.0-9.3)	5.4±2.8	D	
PR091	36	3																																	15	10	7.6 (4.6-10)	7.7±1.6	A	
PR003	2	5																																	15	10	6.3 (4.6-10)	6.4±1.5	A	
PR045	1	5																																	16	10	8.6 (5.3-10)	8.2±1.9	D	
PR008	2	0																																	20	4	2.5 (1.0-7.9)	3.7±3.1	D	
PR029	50	0																																	21	10	10 (5.3-10)	9.4±1.5	B2	D
PR015	2	2																																	14	6	5.6 (2.0-7.6)	5.1±2.1	D	
PR102	15	0																																	9	10	10 (5.6-10)	8.8±1.9	A	
PR040	59	0																																	19	10	10 (10-10)	10±0	B2	
PR043	59	1																																	19	10	10 (6.3-10)	9.4±1.4	B2	
PR004	2	4																																	16	10	10 (6.3-10)	9.2±1.4	D	
PR019	11	7																																	15	9	7.9 (1.0-9.3)	7.1±2.4	B1	E
PR142	29	2																																	13	10	10 (7.3-10)	9.7±0.9	B1	
PR038	59	0																																	19	10	6.3 (5.3-10)	6.7±1.8	D	
PR039	49	3																																	13	10	10 (5.3-10)	9.1±2.0	D	
PR115	21	3																																	14	10	10 (10-10)	10±0	B2	F
PR137	3	0																																	12	10	6.8 (5.6-10)	7.5±1.8	D	
PR118	36	5																																	12	8	4.7 (0-8.6)	4.6±3.0	A	
PR020	65	3																																	11	7	7.8 (1.0-10)	6.9±3.5	A	G
PR064	10	0																																	11	10	9.6 (7.3-10)	9.3±0.9	D	H
