# Supplementary figures and images for: Genotypes and Pathogenicity of Cellulitis Isolates Reveal Traits That Modulate APEC Virulence
Source: PLoS One. 2013 Aug 19;8(8):e72322. doi: 10.1371/journal.pone.0072322 (PMC3747128; doi:10.1371/journal.pone.0072322)

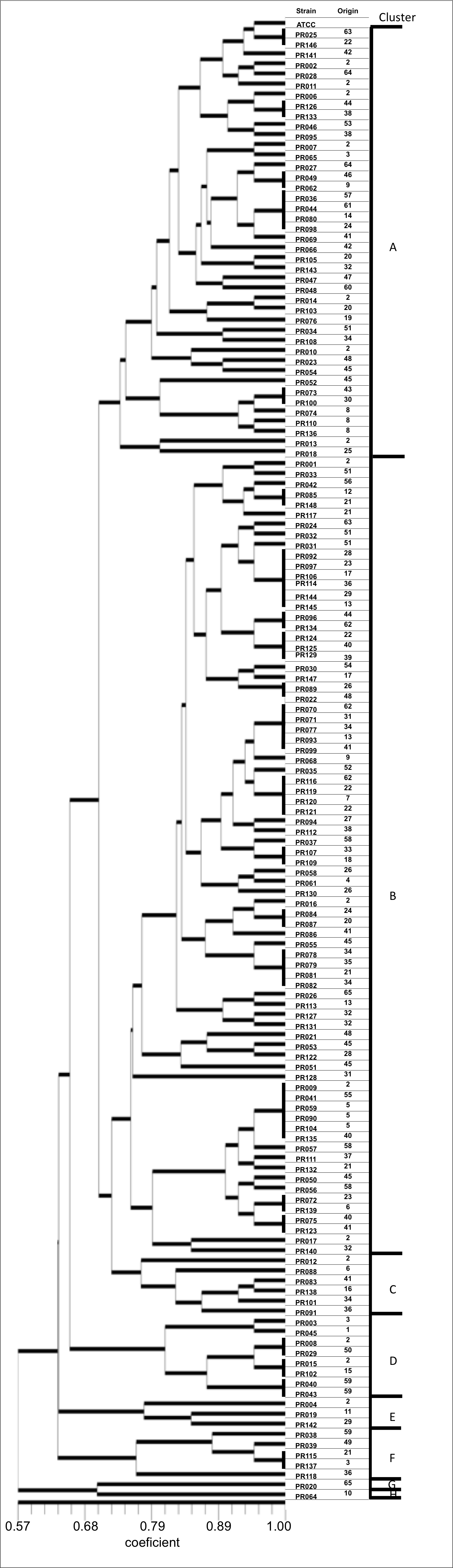

Supplement: Figure S2 — ARDRA profile of 144 APEC isolates. The ARDRA dendrogram was constructed by UPGMA based upon enzyme restriction digestion of amplified 16-23S DNA intergenic spacer regions. The column Strain shows isolate designation; the column Origin, source of isolate, with 1 to 65 designating each of the 65 farms from which the isolates were collected. Cluster designates the 8 genotypic clusters (A to H) into which strains with 80% similarity were grouped. E. coli ATCC25922 was analyzed as a reference strain. (TIF) [file pone.0072322.s002.tif]
